# An Evolutionary Mosaic Challenges Traditional Monitoring of a Foundation Species in a Coastal Environment—The Baltic *Fucus vesiculosus*

**DOI:** 10.1111/mec.17699

**Published:** 2025-02-17

**Authors:** Ricardo T. Pereyra, Alexandra Kinnby, Alan Le Moan, Olga Ortega‐Martinez, Per R. Jonsson, Stefania Piarulli, Matthew I. M. Pinder, Mats Töpel, Pierre De Wit, Carl André, Halvor Knutsen, Kerstin Johannesson

**Affiliations:** ^1^ Department of Marine Sciences Tjärnö Marine Laboratory, University of Gothenburg Strömstad Sweden; ^2^ Department of Climate and Environment SINTEF Ocean Trondheim Norway; ^3^ Department of Marine Sciences University of Gothenburg Gothenburg Sweden; ^4^ IVL Swedish Environmental Research Institute Gothenburg Sweden; ^5^ Department of Biological and Environmental Sciences University of Gothenburg Gothenburg Sweden; ^6^ Center for Coastal Research, Department of Natural Sciences University of Agder Kristiansand Norway; ^7^ Institute of Marine Research Flødevigen Norway

**Keywords:** demographic inference, foundation species, *Fucus radicans*, genetic monitoring, marginal habitat, partially clonal species, postglacial sea, SNP panel

## Abstract

During periods of environmental change, genetic diversity in foundation species is critical for ecosystem function and resilience, but it remains overlooked in environmental monitoring. In the Baltic Sea, a key species for monitoring is the brown seaweed *Fucus vesiculosus* , which forms sublittoral 3D habitats providing shelter and food for fish and invertebrates. Ecological distribution models predict a significant loss of Baltic *F. vesiculosus* due to ocean warming, unless populations can adapt. Genetic variation and recombination during sexual reproduction are essential for adaptation, but studies have revealed large‐scale clonal reproduction within the Baltic Sea. We analysed genome‐wide single nucleotide polymorphism (SNP) data from the east Atlantic, the “Transition zone,” and the Baltic Sea, and found a mosaic of divergent lineages in the Baltic Sea, contrasting an outside dominance of a few genetic groups. We determined that the previously described endemic species *Fucus radicans* is predominantly a large female clone of *F. vesiculosus* in its northern Baltic distribution. In the two Estonian sites, however, individuals earlier referred to as *F. radicans* are sexually and reproductively isolated from Baltic *F. vesiculosus* , revealing a separate lineage that may have diverged long before the formation of the Baltic Sea. Monitoring Baltic *Fucus* without considering this genetic complexity will fail to prioritise populations with adaptive potential to new climate conditions. From our genomic data, we can extract informative and diagnostic genetic markers that differentiate major genetic entities. Such a SNP panel will provide a straightforward tool for spatial and temporal monitoring and informing management decisions and actions.

## 1 Introduction

Adaptation of species during the Anthropocene must be rapid to prevent extinctions, and for multicellular organisms that can rarely rely on new mutations, standing genetic variation is essential (Barrett and Schluter 2008). Indeed, sexually reproducing multicellular species have been shown to adapt to immediate environmental changes (Lescak et al. 2015; Jain and Stephan 2017; Roberts Kingman et al. 2021; Brennan et al. 2022; Garcia Castillo et al. 2024; Nosil et al. 2024) utilising single‐site polymorphisms (single nucleotide polymorphisms [SNPs]) and/or structural variation polymorphisms (Merot et al. 2020). Rapid adaptive changes, however, are often associated with large demographic losses, potentially leading to extinction due to stochastic events during population bottlenecks (Bürger and Lynch 1995).

Despite this critical role of genetic variation, it is rarely considered in monitoring and conservation (Laikre 2010; Sandström et al. 2019). Knowledge of the species' genetic variation and architecture will also prove pivotal with increasingly sophisticated approaches for restoring foundation species. For example, currently ongoing approaches of assisted gene flow or assisted evolution (Nitschke et al. 2024) might become common to actively restore lost genetic variation in key populations (van Oppen et al. 2015; Prieto‐Benítez et al. 2021).

Many habitat‐forming marine species, such as macroalgae (seaweeds), marine plants, and tropical and cold‐water corals, are partially clonal and exhibit more complex spatial distributions of their genetic diversity than fully sexual species (Kliber and Eckert 2005; Dahl et al. 2012; Arnaud‐Haond et al. 2020; Ries et al. 2023; Krueger‐Hadfield 2024). For example, stochastic processes during range expansion can temporarily remove sexual reproduction (Rafajlović et al. 2017), significantly impacting the adaptive potential of a species. In the resulting clonal lineages, however, somatic mutations that segregate among numerous vegetative offspring can provide a source of variation for selection to act upon (Reusch et al. 2021). Over time and in the absence of recombination, however, somatic mutations and the stochastic loss of genotypes will lead to an accumulation of deleterious mutations (“Muller's ratchet”; Butlin 2002; Berdan et al. 2021). Furthermore, fixation of positive new mutations will be slow due to linked genetic variation with low fitness (“the Hill‐Robertsson effect”; Felsenstein 1974). These mechanisms markedly reduce the potential for rapid adaptation to changing environments in predominantly clonal populations.

The Baltic Sea is a postglacial, semi‐isolated brackish‐water environment with a narrow entrance from the Atlantic that reduces water exchange and prevents tidal ventilation. In semi‐enclosed coastal gulfs like the Baltic Sea, climate‐related changes in physical factors will occur significantly faster than in the large oceans (Reusch et al. 2018). Hence, by the end of the century, warming is expected to increase sea temperature by 3°C–6°C and reduce salinity and transparency (Gröger et al. 2019). The already low salinity (3‰–12‰) has led populations of marine species that colonised the Baltic Sea approximately 8000 years ago to tolerate or adapt to these conditions, as evidenced by genetic and phenotypic divergence from Atlantic populations (Johannesson and André 2006; Johannesson et al. 2020). A few species have evolved strong barriers and are currently completely isolated from Atlantic populations (Momigliano et al. 2018; Hemmer‐Hansen et al. 2019; Helmerson et al. 2023). Some other species diverged already long before the opening of the Baltic Sea, and with one lineage inside and the other outside the Baltic Sea, these old barriers continue to support the isolation of the Baltic Sea populations (Riginos and Cunningham 2005). For all marine Baltic populations, the predicted rapid effects of climate change will exert significant pressure on top of the current constraints of living in an isolated and marginal environment.

The brown macroalgae, *Fucus vesiculosus* , is common in coastal Atlantic waters (Hatchett et al. 2022) and is the only large brown macroalgae over much of the Baltic Sea. Here, it forms a three‐dimensional habitat from 0.5 to 10 m depth for small invertebrates and fish (Wikström and Kautsky 2006). Baltic populations of *F. vesiculosus* are adapted to constant submersion (Pearson et al. 2000), and exhibit higher growth rates in low salinity than in marine conditions (Bäck et al. 1992; Johansson et al. 2017). Predicted environmental conditions for the next 70–80 years may result in the loss of *F. vesiculosus* in the northern and eastern Baltic Sea (Jonsson et al. 2018; Kotta et al. 2019) unless populations can adapt to the new conditions. However, the adaptive potential of *Fucus* in the northern and eastern Baltic populations is hampered by the loss of sexual function due to polyspermy (Serrão et al. 1999) and by the replacement of sexual recruitment by asexual reproduction in many populations (Bergström et al. 2005; Tatarenkov et al. 2005). For example, in the Gulf of Bothnia, a widely distributed and presumably very old clone appears to have formed during the species' early expansion into the Baltic Sea (Ardehed et al. 2016; Rafajlović et al. 2017; Pereyra et al. 2023). Nonetheless, there are also sexually recruiting populations in the Gulf of Bothnia and Gulf of Finland in very low salinities (< 4‰; Ardehed et al. 2016), resulting in a highly complex population genetic structure.

In this study, we address the relevance and utility of incorporating information on genetic diversity into management and conservation practices, using the Baltic *Fucus* as a case study. While our findings are specific to *F. vesiculosus* , the broader insights are applicable to other partially clonal species and coastal ecosystems. We benchmark the potential of genetic data in monitoring programs, comparing it to traditional ecological monitoring methods based on the phenotypic recognition of taxa. Additionally, we investigate whether ecological monitoring can effectively identify the key evolutionary lineages of the Baltic *Fucus*. We also examine the importance of high spatial resolution of the geographic sampling and knowledge about connectivity patterns of populations for conservation purposes. We show that a limited number of sites representing major distribution areas do not suffice to establish a monitoring baseline. Furthermore, we illustrate how the use of thousands of genome‐wide genetic markers provides significant advantages for conservation guidance compared to older genetic methods, such as microsatellites and single‐locus markers. Finally, we propose the use of an informative and diagnostic SNP panel as a relatively inexpensive and simple method to identify key genetic entities within species once a comprehensive baseline investigation is available. For partially clonal species, we show how clonal richness (*R*), inbreeding coefficient (*F*_IS_), and linkage disequilibrium (LD) can be used to illustrate and communicate the potential for evolutionary change in populations within conservation and management contexts.

## 2 Materials and Methods

### 2.1 The Study Organisms

#### 2.1.1 Reproductive Biology and Morphology of *F. vesiculosus*

The species is dioecious, and individuals release either eggs or sperm into the water column. Fertilised eggs are negatively buoyant and settle within metres of the parent individuals (Serrão et al. 1997). This leads to limited dispersal, although dislodged plants with reproductive tissue may release gametes after being drifted to new places. As evident from the genetic data, the species is subdivided into genetically discrete local populations (Tatarenkov et al. 2007; Ardehed et al. 2016; Rinne et al. 2018; Pereyra et al. 2023). In addition to sexual reproduction, adventitious branches can detach, reattach to the substratum, and grow as new individuals (Tatarenkov et al. 2005). Initially, it was suggested that this asexual strategy was unique to the brackish‐water populations within the Baltic Sea (Tatarenkov et al. 2005). However, recent findings show that the species is also partially clonal along the Danish coasts, albeit to a lesser extent than in the Baltic Sea (Pereyra et al. 2023). Notably, individuals grown from reattached adventitious branches also develop receptacles where gametes are formed. In younger clonal lineages, these gametes might remain functional, whereas, in older clonal lineages this functionality could be lost (Rafajlović et al. 2017). The formation of receptacles in both clonal and sexually reproducing individuals makes it challenging to distinguish between sexually and asexually recruited individuals in the field. There is also individual variation in *F. vesiculosus* in reproductive season (Berger et al. 2001), lifestyle (free‐floating vs. attached individuals; Preston et al. 2022), and morphology (Kinnby et al. 2019). Thallus shape, for example, may change dramatically over environmental gradients and become smaller and bushier in marginal environments, such as saltmarshes and low‐salinity habitats (Ruuskanen and Bäck 1999, 2002; Sjøtun et al. 2017). Hybridisation with closely related species has also been reported, and it sometimes contributes to increased phenotypic divergence (Sjøtun et al. 2017).

#### 2.1.2 The Enigmatic *Fucus radicans*

The Baltic taxon *Fucus radicans* Bergström & Kautsky was recently described as a distinct species, characterised by its narrow‐fronded, dwarf, and bushy thallus, and consistent differences at microsatellite loci compared to sympatric *F. vesiculosus* (Bergström et al. 2005). *F. radicans* was initially identified along the Swedish and northern Finnish coasts of the Gulf of Bothnia (Pereyra et al. 2009; Johannesson et al. 2011; Rinne et al. 2018). Later, its presence was also suggested at two sites in the Estonian archipelago (Forslund and Kautsky 2013). However, *F. radicans* remained an enigmatic species. For example, it was still unclear whether morphologically bushy and small individuals along the southern and eastern coasts of Finland were *F. radicans* or *F. vesiculosus* (Ardehed et al. 2016; Rinne et al. 2018). Additionally, genetic analyses using microsatellites suggested a complex and potentially parallel origin of the Swedish and Estonian populations (Pereyra et al. 2013). Finally, *F. radicans* is a putative endemic species (Pereyra et al. 2009; Schagerström and Kautsky 2016), and determining the origin and contemporary relationship between *F. radicans* and Baltic *F. vesiculosus* , and elucidating the relationship between the geographically disparate populations of *F. radicans* (Gulf of Bothnia and the Estonia islands of Saaremaa and Hiiumaa) has remained important.

### 2.2 Sampling

**FIGURE 1:**
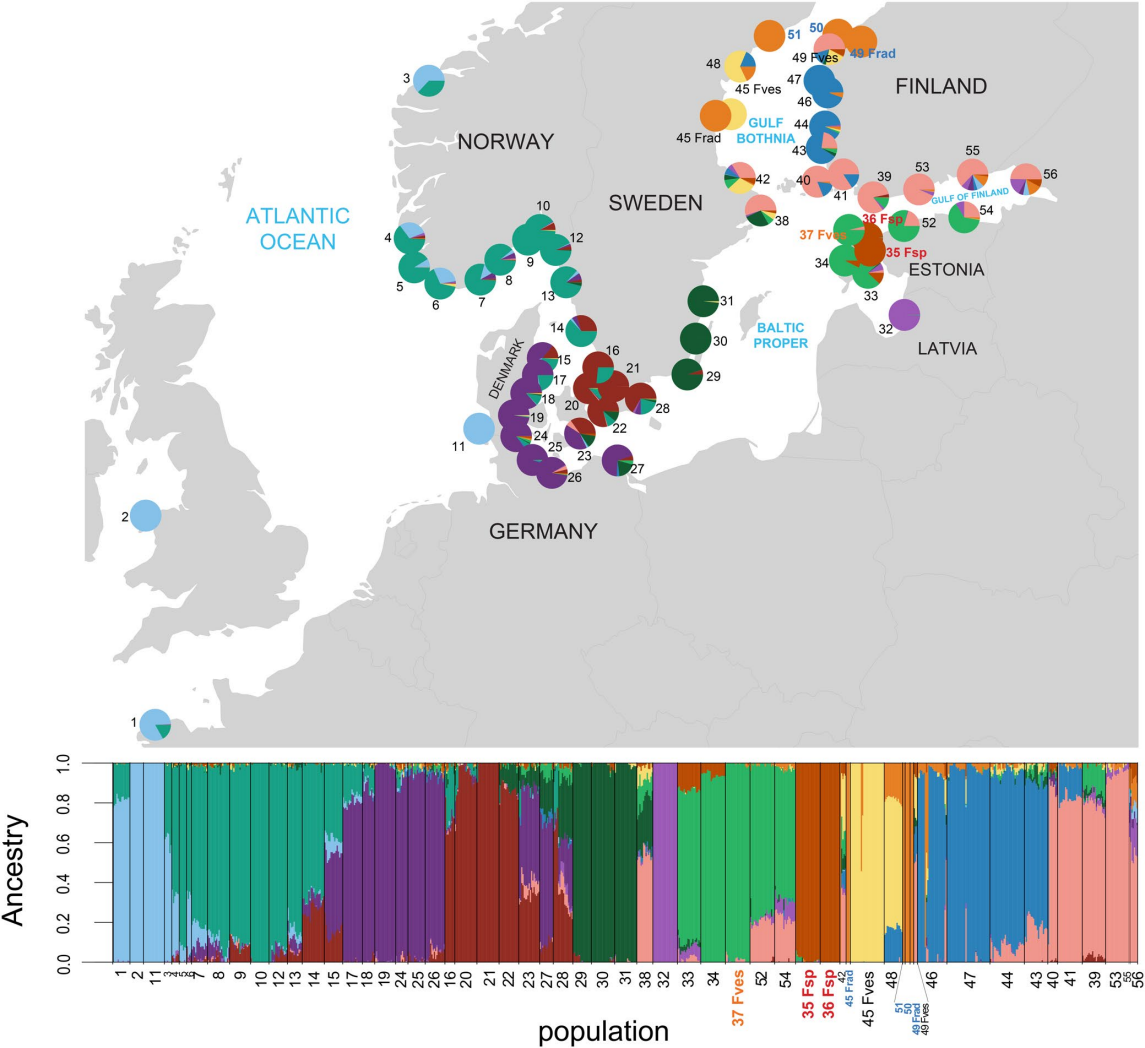
Genetic ancestry analysis including all individuals from the 55 sampled sites subdivided into *K* = 12 genetic clusters. The map illustrates the geographic distribution of these clusters based on the estimated individual ancestry from the genetic admixture plot below. Please note that some sites where all the sampled individuals are part of clonal lineages (one to three clones) render very thin genetic clusters in the admixture plot due to the inclusion of only one individual per genotype (e.g., sites 45, 49–51, and 55; see methods for details of samples included). Also note that sites 36 and 37 are completely sympatric but individuals are kept separate for the sake of the ancestry analysis.

Between 15 and 20 individuals (in some cases fewer; see Table S1) were sampled from 55 sites representing the distribution of *F. vesiculosus* (including *F. radicans* ) throughout the Baltic Sea (sites 28–55, Figure 1), the Transition zone (Danish sounds and Kattegatt, sites 14–27), and nearby Atlantic waters (sites 1–13), with Roscoff (site 1), Bangor (site 2), and Ålesund (site 3) being the most distant sites. At the locality Sarve, Estonia, the morphotype previously referred to as *F. radicans* , but here referred to as (*Fucus* sp.), coexists sympatrically with *F. vesiculosus* . These two entities are represented separately as site 36 (*Fucus* sp.) and site 37 ( *F. vesiculosus* ), respectively. From southeast Norway and further into the Baltic Sea, the spatial coverage is approximately one sampling site per 100–200 km coastline. The species is essentially absent along the coasts of eastern Germany and Poland, and these areas were not visited. All sampled individuals were photographed on a flat background in the field before four to six tips per individual were retained in labelled zip‐lock bags and brought to the laboratory for DNA extraction.

### 2.3 Morphological Analysis

One aim of our study was to determine if morphology (as currently used in monitoring) also reflects genetic ancestry and population structure. We used three morphometric characters: Frond width (FW), distance between dichotomies (DBDs), and undulation index (UX). The first two characters were used as diagnostic to differentiate *F. radicans* from *F. vesiculosus* (Bergström et al. 2005; Pereyra et al. 2013) but may vary due to local environmental conditions (e.g., wave exposure, salinity; Bergström et al. 2005; Ruuskanen and Bäck 2002). The UX was calculated as the ratio between the marginal undulation of a given thallus branch and the length of a straight line along the same branch (Broisat et al. 2011). Each individual was characterised using the average of three measurements taken from different branches with the image analysis software ImageJ64 (Schneider et al. 2012). Principal component analysis (PCA) on the morphometric measurements was performed in RStudio (v.2024.04.1 + 748) using the package “ggfortify” v.0.4.17. One PCA was conducted using all individuals from seven populations in the Gulf of Bothnia and all six Estonian populations, covering areas where *F. radicans* has previously been found or could potentially be present. A separate PCA was also conducted for only the six populations from Estonia. A dendrogram for the morphological data was created using the R package “vegan” v.2.6.4, after which the grouping was compared with a grouping based on genetic distances (see Section 2.5).

### 2.4 Genomic Data, Identification of Clones, and Assessing Evolutionary Potential

DNA extractions and individually barcoded 2b‐RAD libraries followed the procedures in Pereyra et al. (2023). The libraries were sequenced using the Illumina Novaseq6000 platform at SciLifeLab, Uppsala. A total of 962 individuals from 55 different sites were sequenced (Figure 1). Removal of PCR duplicates, quality filtering, and variant calling were performed on the computer cluster Albiorix (M. Töpel, IVL, Sweden), following a reference‐based pipeline modified from Mikhail Matz available at https://github.com/z0on/2bRAD_GATK. Reads were mapped to a *F. vesiculosus* draft genome assembly previously used for population genomic studies (Kinnby et al. 2020; Pereyra et al. 2023; NCBI Bioproject No. PRJNA629489). Variant calling calibration was carried out with technical replicates, and the genotyping accuracy based on the overall match between technical replicates was 95.02%, which was used as a preliminary threshold to identify clones. After variant recalibration, data were further filtered for poor coverage and > 5% data missingness on loci and individuals. The final dataset included only bi‐allelic, polymorphic loci, rendering a total of 37, 302 SNPs. The effect of missingness was examined by assessing individual heterozygosity in relation to the amount of missing data within each population and across the overall dataset to identify individuals with outlier heterozygosity values potentially due to missing data. Following this assessment, no additional data were removed.

We identified clones (repeated genotypes = ramets) using a genetic distance‐based approach (Kamvar et al. 2014, 2015), as detailed in Pereyra et al. (2023). Importantly, we used technical replicates to aid the clone identification by producing (nearly) identical genotypes, after accounting for sequencing errors. Subsequently, we estimated a genetic distance cut‐off threshold to distinguish clones (genetic distance = 0.0279) using the “cutoff predictor” function in the R package “Poppr” v.2.9.3 (Kamvar et al. 2015). Using this threshold, we identified similar multi‐locus genotypes (MLGs = genets) that resulted in 818 contracted MLGs in 962 total genotyped individuals, after removal of technical replicates (details in Pereyra et al. 2023). The genetic distance‐based method used to detect clonality is expected to show, from a distribution of genetic distances among individuals, distinct peaks towards low distances (instead of a strict unimodal distribution), revealing high genotypic similarity of MLGs originating from the same reproductive event (clones) (Douhovnikoff and Dodd 2003; Meirmans and Van Tienderen 2004; Arnaud‐Haond et al. 2007).

In partially clonal species, such as *F. vesiculosus* , recombination through sexual reproduction is critical for adaptation under climate change. Linkage disequilibrium (LD) reveals the extent of non‐random associations of loci due to lack of recombination, and clonal richness (R) is an index of genotypic diversity that results from the balance between recombination of new genotypes and loss of diversity by natural selection and genetic drift. In addition, the inbreeding coefficient, *F*_IS_, describes the genetic variation based on heterozygosity, where in a large recombining population it is expected to be close to zero, while negative values will indicate the presence of clonality (Balloux et al. 2003; Arnaud‐Haond et al. 2020; Stoeckel et al. 2021). We assessed the extent of recombination in each locality by estimating LD using the standardised index of association ($\bar{r}$_d_) in “Poppr” (Brown et al. 1980; Agapow and Burt 2001). An index of association close to zero is expected in recombining populations through sexual reproduction, as opposed to clonally reproducing demes (Kamvar et al. 2015). However, performing genome‐wide genotyping at thousands of loci will likely render some non‐random loci associations (i.e., LD) despite complete sexual random mating in each population. Still, LD is expected to be relatively higher in clonal than in sexually reproducing populations. For clonal‐richness, we used R = (*G* − 1)/(*N* − 1), where *G* is the number of genotypes and *N* is the number of sampled individuals (Dorken and Eckert 2001). The inbreeding coefficient *F*_IS_ was calculated for each locality using the R package “radiator” v.1.1.2 (Gosselin et al. 2020). Estimates of clonal richness, inbreeding coefficient, and LD were performed, including all individuals per site (sexually recruited and clones) to capture the diversity relative to the number of individuals as well as to estimate the non‐random association of loci due to clonality.

### 2.5 Individual Genetic Relationships

We calculated pairwise genetic distances among individuals using the function diss.dist in Poppr, which is a dissimilarity measure based on the percentage of allelic differences (Kamvar et al. 2014). We used all individuals per site (sexually recruited and clones) for these estimates and using the genetic distance matrix generated, we plotted a Neighbour‐joining dendrogram with bootstrap support to illustrate the large clonal genetic clusters as well as the genetic similarities among individuals.

We performed a PCA using the package “dartR” v2 (Mijangos et al. 2022) to illustrate the global genetic differentiation relative to the sampling geography. We further estimated the individual ancestry proportions using the “snmf” function of the R package using “LEA” v3.8.0. (Frichot and François 2015). For both the PCA and the individual ancestry analysis, we removed all genotype copies (ramets) within each locality, leaving only one individual per MLG (genet) to avoid biases due to repeated genotypes while still capturing the genotype diversity within each locality.

### 2.6 Demographic History Reconstruction

We investigated the origin of the Estonian *Fucus* populations present in Sarve (sites 36 and 37 designated to *Fucus* sp. and *F. vesiculosus* , respectively) and Pulli Pank (site 35) earlier referred to as *F. radicans* (Forslund and Kautsky 2013). In contrast to Gulf of Bothnia *F. radicans* , the Estonian individuals are sexually recruited and morphologically and genetically divergent from sympatric *F. vesiculosus* (Pereyra et al. 2013). In Sarve, the two entities co‐exist, and these populations were used to analyse their relationship using demographic inference. No ramets were included in this analysis, as only sexually recruited individuals were found in these localities. We used the folded version of the joint Site Frequency Spectrum (jSFS) and diffusion approximation, as implemented in the software δaδi (Gutenkunst et al. 2009). We compared the ability of 22 contrasted scenarios to reproduce our observed pattern of genome‐wide divergence in the analysed SNPs. The scenarios were divided into four basic divergence histories: Strict Isolation (model SI with four variations: SI, SIG, SI2N, SI2NG); Isolation with continuous Migration (model IM with six variations: IM, IMG, IM2N, IM2m, IM2NG, IM2mG); isolation with Ancestral Migration (model AM with six variations: AM, AMG, AM2N, AM2m, AM2NG, AM2mG); and Secondary Contact (model SC with six variations: SC, SCG, SC2N, SC2m, SC2NG, SC2mG). The variations of all the basic scenarios include an exponential population growth (model G) that can be asymmetrical in the two lineages, and the Hill‐Robertson effect captured by fitting two effective population sizes affecting a proportion Q and 1‐Q of the genome (model 2N). In addition, for the scenario of divergence with gene flow (IM, SC, and AM), the models also included the possibility of heterogeneous migration rate along the genome (2m model) as expected at an intermediate stage of speciation. All the models included variations of effective population size in the ancestral population, as suggested by Momigliano et al. (2021). More details about the different models and their inferences are available in Rougeux et al. (2019). All models were adjusted to the observed jSFS 20 times using a three‐step optimisation procedure described in Tine et al. (2014). Each fit was then compared using the Akaike information criterion (AIC) and a very conservative threshold; only those models that had ΔAIC< −10 were considered as stronger models than others. As the parameters inferred from δaδi are scaled to the ancestral population mutation rate (ϴ), these were transformed into biological parameters using a generation time of 4 years (Malm and Kautsky 2004) and a base pair mutation rate of 1 × 10^−8^.

### 2.7 Biophysical Model of Seascape Connectivity

Genetic structure is strongly influenced by barriers to gene flow within the seascape. We estimated current opportunity for gene flow with a biophysical model combining an ocean circulation model with particle tracking to simulate *Fucus* dispersal. The relative importance of different mechanisms of dispersal for *F. vesiculosus* is largely unknown, where spores disperse very short distances (Serrão et al. 1997), while dislodged and drifting individuals may deliver spores or gametes over long distances (Rothäusler et al. 2020). Here dispersal was modelled assuming drift in surface waters with a duration of 10–20 days, and with spawning between May and October. As an estimate of gene flow, we calculated the multi‐generation connectivity across 32 generations assuming stepping‐stone dispersal within the seascape (Jahnke and Jonsson 2022). The part of the Baltic Sea seascape with potential habitat for *F. vesiculosus* was predicted with a species distribution model. Finally, multi‐generation connectivity between 50 of the sample sites, excluding the five most distant Atlantic sites (1–5), was presented as a heatmap (further details are included as Supporting Information).

## 3 Results

### 3.1 Genetic Structure, Connectivity, and Evolvability of Baltic *Fucus*

**FIGURE 2:**
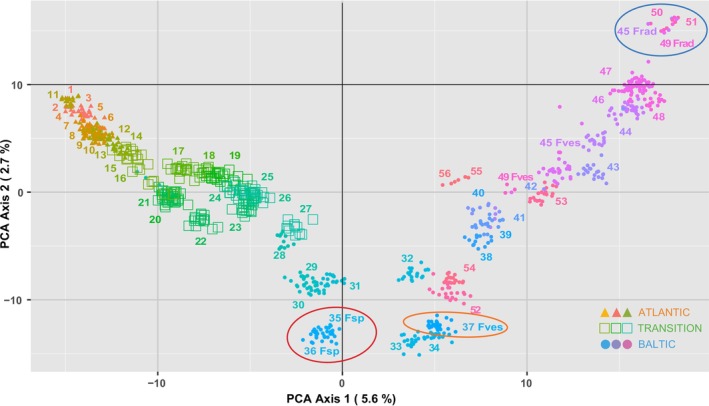
A principal component analysis (PCA) of all individuals from the 55 sites. Colour gradients and shapes indicate their geographic position, from the Atlantic (orange‐gradient triangles), across the Transition zone (green‐gradient squares), the southern Baltic Sea (blue‐gradient circles), and the northern and eastern Baltic Sea (purple and pink circles). Large red ellipse encloses individuals of the new *Fucus* sp. present in two Estonian sites (35 and 36). Large orange ellipse encloses individuals of *F. vesiculosus* sympatric with *Fucus* sp. in site 36 but assigned site number “37” (see Figure 1 legend). Large blue ellipse encloses individuals of *Fucus radicans* (sites 45, 49–51).

Our sampling covered the complete distribution of *F. vesiculosus* within the Baltic Sea and the Transition zone (Figure 1). The sampling and subsequent analysis generated genetic data from 55 different sites, including reference sites in the nearby Atlantic. A PCA on all SNP data revealed that most Baltic and Transition zone sites were distinct, either individually or in small clusters of nearby sites. Furthermore, this separation corresponded strongly to the geographic axis of separation (Figure 2). In contrast, the Atlantic populations (1–13), including the outermost Transition zone sites (14–15), largely overlapped with no clear geographic structure (Figure 2).

**FIGURE 3:**
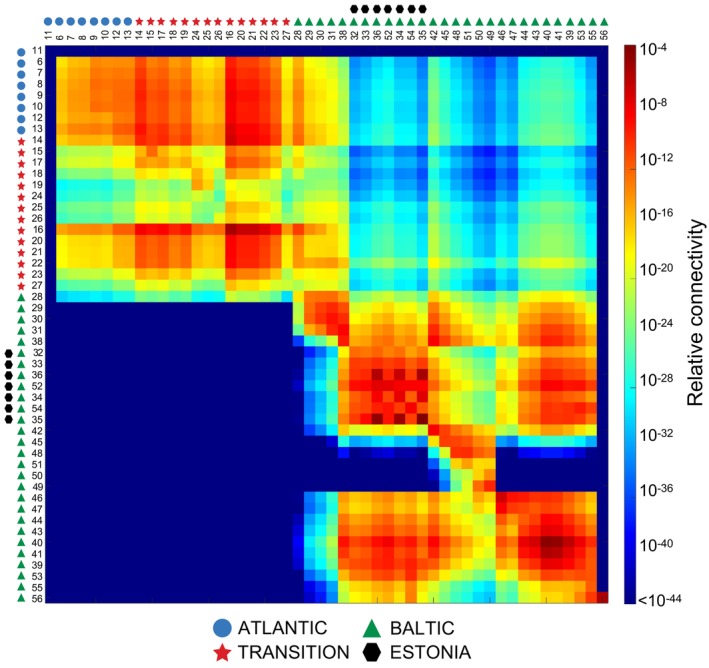
Relative connectivity between localities estimated by a dispersal model driven by oceanographic currents. The matrix indicates the direction from sites in columns to sites in rows. Note that the five most distant Atlantic sites (1–5) are excluded from this analysis.

The individual genetic ancestry analysis showed a subdivision of all sampled individuals into *K* = 12 genetic clusters. The four most distant Atlantic sites formed a single group, while the sites from the Transition zone and the Baltic Sea formed the remaining 11 genetic clusters (Figure 1). For example, within a radius of 200 km involving Estonian and SW Finnish sites, five genetic clusters were represented across only seven sites (32–36, 39, 52). In other areas, one genetic cluster dominated multiple sites along the coast (e.g., sites 29–31), a pattern that mostly corroborated high connectivity based on oceanographic data (Figure 3). In other cases, ocean currents acted as barriers to gene flow, for example, separating sites on either side of the Gulf of Finland and Gulf of Bothnia, or between the Danish and nearby Swedish coasts (Figures 1 and 3). Notably, two Swedish sites (38 and 42), and one Danish site (23) were true melting pots, sharing genetic ancestry with sites in various directions (Figure 1). Again, the oceanographic connectivity results corresponded with these patterns (Figure 3). Conversely, a genetically distinct site in the Gulf of Riga (site 32, Figure 1) appeared well‐connected to nearby sites based on the oceanographic data (Figure 3), but other factors may explain its genetic differentiation. The Russian site (56) appeared to be a sink population, as connectivity from this site to other sites was highly restricted. In contrast, the three Estonian sites 33–36 were strongly interconnected with a high potential of migration. For the Baltic Sea as a whole, connectivity was strongly asymmetric with a much higher potential of gene flow out of the Baltic Sea than into it (Figure 3).

**FIGURE 4:**
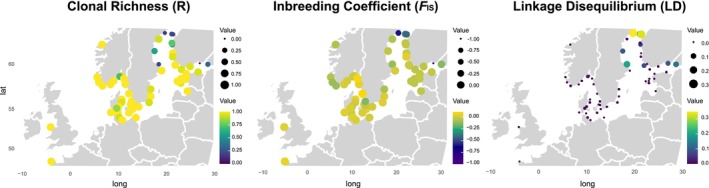
Population estimates of genetic diversity by locality, including clonal richness (R), inbreeding coefficient (*F*_IS_) and linkage disequilibrium (standardised index of association $\bar{r}$_d_), depicting genetic recombination. Higher values of R indicate greater standing genetic variation. Lower negative values of *F*_IS_ indicate increased rates of clonality, while lower values of $\bar{r}$_d_ indicate higher rates of recombination (more sexual reproduction and less cloning). Bubble sizes and colour gradients indicate value scales. Note that each estimate is presented on a distinct value scale. The data points on each map correspond to the same sites as in Figure 1.

High clonal richness (R) indicates a high proportion of different genotypes in each site, and thus greater standing variation. On the other hand, increasingly positive inbreeding coefficients (*F*_IS_) indicate higher levels of inbreeding, while increasingly negative values suggest greater levels of clonality. Low LD indicates sexual reproduction and frequent recombination, which generates new genotypes in a population. These three estimates varied considerably among the sites. In the southern Baltic Sea, the Transition zone, and the Atlantic, sites generally displayed high R, slightly negative but generally close to zero *F*_IS_ and relatively low LD, as expected from populations with almost no clonality. However, a few sites in this area stood out, showing lower R, increasingly negative *F*_IS_, and increased LD, indicators of populations with partial clonality (sites 9, 19 R and LD or even 16 in *F*_IS_; Figure 4). Remarkably, low R, high negative *F*_IS_ and high LD were found in several sites inside the Gulf of Bothnia and the Gulf of Finland, with northernmost and easternmost sites, respectively, being the most extreme. In one Gulf of Finland site, all individuals were the same clone (site 55; R = 0, *F*_IS_ = −1). The most extreme LD was found in the northernmost site (site 51; LD = 0.3), but a few other sites, including two sites on the border to the Baltic Proper (site 40 and 42), showed LD values larger than 0.1, implying substantially reduced recombination rates. Importantly, the northern site 48 in the Gulf of Finland revealed high R, *F*_IS_ close to zero and almost no LD, indicating both high standing variation and high recombination rates through sexual reproduction.

### 3.2 Correspondence Between Morphological and Genetic Clustering

**FIGURE 5:**
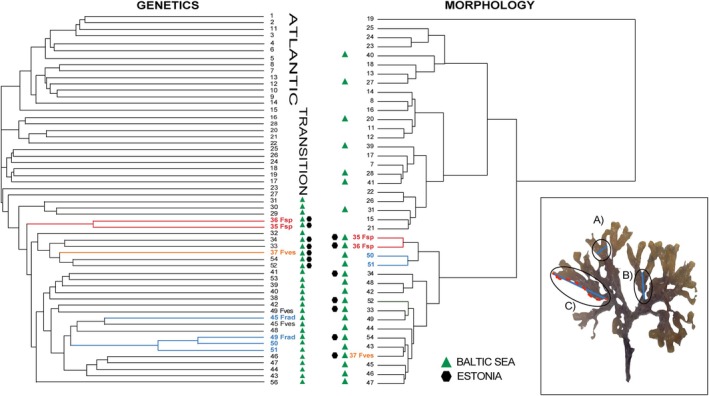
Genetic and phenotypic dendrograms showing the clustering of sites according to genetic affinity and phenotypic similarity, respectively. Sites without icons in the morphology dendrogram belong to clustered Atlantic and Transition zone sites. Site numbers are as in Figure 1. Branches are coloured as *Fucus* sp. (red), *F. vesiculosus* from site 37 (orange), which is sympatric with *Fucus* sp. in site 36 (red), and a large female clone, Frad (“ *F. radicans* ”) (blue). The inset figure illustrates the three morphological measurements taken from each sampled individual: (A) frond width (FW), (B) distance between dichotomies (DBDs), and (C) undulation index (UX), where the red dotted line following the thallus margin and the blue straight line along the same blade illustrate the measurements used to calculate this index.

To test whether ecological monitoring based on the phenotypic traits recognises the same population structure as established from the genetic data, we averaged the morphologies of all individuals within each site from which we performed clustering based on morphology. The resulting overall clustering did not reflect the geographic structure Atlantic‐Transition zone‐Baltic Sea observed in the genetic clustering (Figure 5). Instead, except for one outlier population from an extremely sheltered site (Kolding, site 19), the morphological analysis grouped the sites into two major clusters. One cluster included all Transition zone and Atlantic sites, as well as sites 28–31 from the southern Baltic Proper, and sites 39–41 from the Finnish SW coast. The other cluster contained all the Estonian sites (including those along the Gulf of Finland) and all sites from the Gulf of Bothnia (Figure 1). Within this latter cluster, two of the four sites with individuals previously identified as *F. radicans* in the Gulf of Bothnia grouped with the two Estonian sites (site 35, 36). In the following sections, we demonstrate that this morphological clustering only partly reflects the complex ancestry of the different *Fucus* lineages in the Baltic region.

### 3.3 The Taxonomic Identity and Ancestry of *Fucus radicans*

**FIGURE 6:**
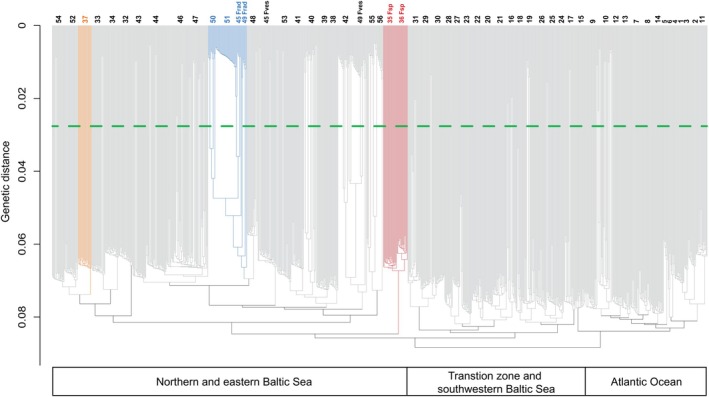
Neighbour‐joining (NJ) tree including all genotyped individuals showing three main groups of *F. vesiculosus* : The Atlantic group (sites 1–15), the Transition zone and SW Baltic Sea (sites 16–31), and northern and eastern Baltic Sea (sites 32–56). Site numbers according to Figure 1. The bushy, narrow fronded Estonian individuals present in sites 35 and 36 (*Fucus* sp.) are marked in red, and the sympatric individuals of *F. vesiculosus* from the same site but numbered as 37 in the ancestry analysis (Figure 1) are shown in orange. The large female clone (“ *F. radicans* ”) is marked in blue. Note the differences in branch lengths indicating sexual (long branches) and asexual recruitment (short branches).

**FIGURE 7:**
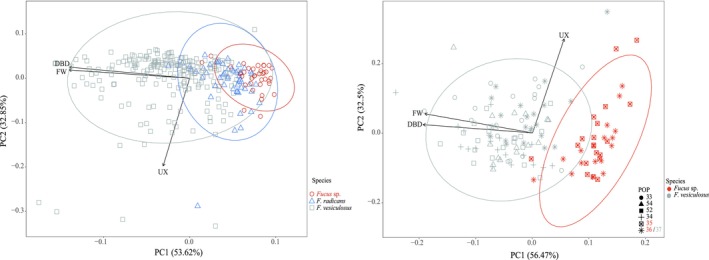
Principal component analysis (PCA) of three morphological traits measured in all individuals from seven Gulf of Bothnia sites (42, 45, 46, 47, 49, 50, 51) (left plot), and six Estonian sites (33–36, 52, 54) (right plot). The three morphological traits are: frond width (FW), distance between dichotomies (DBDs), and undulation index (UX). The individuals belong to three different lineages of *F. vesiculosus* : Baltic *F. vesiculosus* , a large female clone of Baltic *F. vesiculosus* (“ *F. radicans* ”), and *Fucus* sp., a reproductively isolated lineage of *F. vesiculosus* found in two Estonian sites (35 and 36) (see Figure 6).

Our genome‐wide sequencing shows that the *Fucus* individuals from site 51 (Järnäs, the type locality of *F. radicans* ) form a clonal population within the Baltic *F. vesiculosus* cluster (Figure 6). The predominantly very short branch lengths in this population indicate small genetic distances among individuals, consistent with a clonal lineage. Furthermore, this population exhibited low R, strongly negative *F*_IS_ and very high LD, all indicative of low recombination activity (Figure 4). Our data corroborate earlier findings that this clonal lineage is dominant across many sites over 550 km of the Gulf of Bothnia coasts (Ardehed et al. 2015; Pereyra et al. 2023, this study Figure 6). This is a female clone corresponding to the taxon named *F. radicans* . On average, the morphology of this large female clone was characterised by a small frond width and short DBDs, distinguishing it from the average values of these traits in other *F. vesiculosus* individuals (as described in the original study, Bergström et al. 2005). The exception was thallus undulation where there was no clear separation (Figure 7). However, the variation observed within this female clone remained encompassed within the overall variation of *F. vesiculosus* .

Morphologically similar bushy and narrow fronded individuals, also referred to as *F. radicans* in earlier studies (Forslund and Kautsky 2013; Pereyra et al. 2013), were identified at two sites in Estonia (Figure 5, *Fucus* sp.). Unlike the Gulf of Bothnia “ *F. radicans* *”* we found that the narrow fronded Estonian *Fucus* from sites 35 and 36 (Figure 1) were sexually recruited individuals forming a unique and separate lineage from all other Baltic *F. vesiculosus* (Figure 6). This lineage appeared as a sister group to Baltic *F. vesiculosus* , and appears phylogenetically between the Baltic *F. vesiculosus* and *F. vesiculosus* from the Transition zone and the Atlantic (Figure 6). In the Estonian site 36/37 (Sarve), the bushy, narrow fronded *Fucus* coexist in complete sympatry with *F. vesiculosus* but remained genetically isolated in our analysis (Figure 6). Hence, a barrier to gene flow currently maintains the integrity of the two taxa despite overlapping distributions, suggesting that the bushy Estonian individuals belong to a distinct taxon (Figure 6). Henceforth, we refer to this recognisable group of individuals as *Fucus* sp. while acknowledging its uncertain taxonomic rank.

### 3.4 The Origin of *Fucus* sp.

**FIGURE 8:**
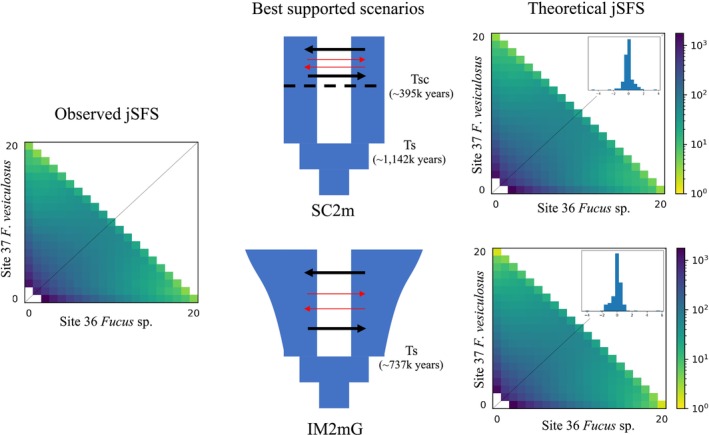
Results of the two best demographic scenarios inferred from δaδi analysis. From left to right, the graphs show the observed joint Site Frequency Spectrum, the schematic representation of the two best demographic scenarios, and the theoretical spectrum inferred from the best demographic scenario (including the distribution of the residual in inset, i.e., jSFS_obs_—jSFS_theo_). The top row shows the result of the Secondary Contact with heterogeneous migration rate across the genome (SC2m), and the bottom row shows the results of the isolation‐with‐migration that also include the heterogeneous migration rate across the genome and an exponential growth of the lineages (IM2mG). In the schematic representation of the two models, the heterogeneous gene flow is expected from lineages with incomplete reproductive isolation and is represented by the two types of arrows, the thin‐red arrow representing reduced gene flow as compared to the neutral one symbolised here by the black arrow.

The two sister groups, *F. vesiculosus* and *Fucus* sp., are both currently established within the Baltic Sea, raising the question of where and when their divergence occurred. One possibility is that they diverged recently within the Baltic Sea, following the initial colonisation of *F. vesiculosus* no more than 8000 years ago after the opening of this postglacial sea. Alternatively, the two taxa could have diverged much earlier, outside the Baltic Sea, and later both colonised this area. Using demographic modelling analysis focused on the two Estonian *Fucus* populations from the sympatric site (36/37), we show that their divergence likely predates the formation of the Baltic Sea. The best‐supported model (SC2m) suggests secondary contact after a period of isolation, while the next best model (IM2mG) proposes divergence under gene flow accompanied by population expansions (Figure 8). In fact, even less likely models support an ancient split of the two lineages long before the opening of the Baltic Sea 8000 years ago (Tables S2 and S3). An ancestral split implies two independent colonisation events into the Baltic Sea long after the divergence of the two sister lineages. Whether this divergence involved a period of isolation or occurred under a scenario with migration remains unclear. The secondary contact scenario with heterogeneous gene flow (SC2m) provided a slightly better fit to the data than divergence under isolation‐with‐migration (IM2mG), with a ΔAIC of six between SC2m and IM2mG. (All model parameters are presented in Tables S2 and S3.)

## 4 Discussion

### 4.1 Complex Population Genetic Structures Are Likely Common in Coastal Areas

Our results show that the population genetic structure of *F. vesiculosus* can be highly complex. This likely reflects the demographic history during periods of range contraction or expansion interacting with contemporary connectivity and selection across local environmental gradients. Such complexity is not unique to *F. vesiculosus* but appears common in coastal populations of both mobile and sessile species inhabiting marine environments (Berg et al. 2015; Momigliano et al. 2018, 2021; Morales et al. 2019; Han et al. 2020; Johannesson et al. 2020; Seljestad et al. 2020; Geburzi et al. 2022; Knutsen et al. 2022; Matschiner et al. 2022; Ries et al. 2023). Repeated migrations of marine species from the Pacific into the northern Atlantic have contributed to this complexity in several taxa (Laakkonen et al. 2020). For example, the bivalve taxa *Mytilus* and *Limecola*, spread from the Pacific into the northern Atlantic during both ancient and recent events (Riginos and Cunningham 2005; Väinölä and Strelkov 2011; Luttikhuizen et al. 2012). Currently, they appear as distinct but hybridising lineages, as seen in the Baltic Sea.

In *F. vesiculosus* , genetic complexity increases from the Atlantic through the Transition zone and into the Baltic Sea (Figure 1). This may be partly due to selection along the salinity gradient, as evidenced by a strong genetic shift in one genomic region including the gene for a calcium‐binding protein (Kinnby et al. 2020). Additional divergence likely stems from stochastic processes during range expansion into the newly opened Baltic Sea (Rafajlović et al. 2017; Pereyra et al. 2023).

### 4.2 Genome‐Wide Markers and Spatially Dense Sampling Resolves Baltic *Fucus* Complexity

Investigations of Baltic *Fucus* using a few microsatellite markers identified large clones (Tatarenkov et al. 2005, 2007; Pereyra et al. 2009; Ardehed et al. 2015; Rinne et al. 2018; Preston et al. 2022), but these studies lacked genetic and spatial resolution to resolve the genetic complexity and evolutionary relationship of the Baltic *Fucus* lineages and the status of *F. radicans* (Pereyra et al. 2013; Ardehed et al. 2016). Genome‐wide markers have recently revealed that asexual recruitment occurs even in the Transition zone, and that large Baltic Sea clones evolved independently multiple times from sexual populations of *F. vesiculosus* (Pereyra et al. 2023). We now show that clonality extends to Norwegian sites, suggesting it is an ancestral trait. We also demonstrate that *F. radicans* in the Gulf of Bothnia is a large clone of *F. vesiculosus* , while the Estonian *Fucus* sp. (earlier referred to as *F. radicans* , Forslund and Kautsky 2013; Pereyra et al. 2013) constitutes a distinct lineage likely diverged from the Baltic *F. vesiculosus* lineage long before the Baltic Sea's formation. Although part of this divergence may be overestimated due to the partial clonality of the species that is not accounted for in the current demographic models, our findings support that the divergence predates the formation of the Baltic Sea by more than an order of magnitude. This suggests that the Baltic Sea was colonised by two independent *Fucus* lineages (similar to the processes discussed above in *Mytilus* and *Limecola*).

The demographic analysis suggested ongoing gene flow between *F. vesiculosus* and *Fucus* sp. in the sympatric site in Estonia, but we found no hybrids and the reproductive seasons of the two linages are nearly completely separated (Forslund and Kautsky 2013). This contrasts with the situation between the Atlantic and Baltic *F. vesiculosus* lineages, which may form a contact zone between sites 31 and 38 along the Swedish coast (Figures 2 and 6) but overlap in reproductive season. Further sampling of the northern Atlantic and Pacific regions is required to clarify these issues and establish a valid taxonomy of this species complex.

### 4.3 The Genetic Diversity of Baltic *Fucus* Needs Adequate Management

Regardless of the historical and contemporary processes shaping the genomic complexity of Baltic Sea and Transition zone *F. vesiculosus* , current data support some immediate conservation actions. The most urgent priority is protecting the rare Estonian taxon (*Fucus* sp.). A second priority is identifying and protecting sexually recruited populations in the Gulfs of Bothnia and Finland, which can only be identified using genetic markers, as clones also develop receptacles and gametes (Tatarenkov et al. 2005). Specifically, discriminating between the large female clone (“ *F. radicans* ”) and sexually reproducing *F. vesiculosus* individuals in the Gulf of Bothnia coast based only on morphological traits is not reliable (Figure 7). While the high population genetic complexity of Baltic *Fucus* will continuously pose challenges for management, oceanographic connectivity modelling can guide genetic sampling towards critical areas. Our data show that the oceanographic connectivity patterns align with the overall population genetic structure, offering a useful tool for conservation planning.

### 4.4 How to Manage Genetic Complexity in the Near Future

While high population genetic complexity poses management challenges, it also provides a larger pool of standing genetic variation that can be crucial for climate adaptation. It is essential to document the distribution of this variation, describe the genetic structure, and establish an appropriate monitoring program (Schwart et al. 2007). Recent rapid approaches based on population census offer a cost‐effective approximation to genetic diversity and can be applied instantly and globally (Hoban et al. 2024; Mastretta‐Yanes et al. 2024). However, in partially clonal species, non‐genetic approaches cannot discriminate clonal from sexual populations, a critical factor for management. To address this, an initial mapping step using high‐resolution genome‐wide markers and a dense geographic sampling is needed. With a baseline in place, genetic monitoring strategies can be simpler and less costly, focused on recombination rates and standing genetic variation, enabling the prioritisation of populations with the highest adaptive potential. In *F. vesiculosus* , recombination potential varies widely, even among nearby populations, and it is critical to capture these differences in management. Monitoring should focus on three indicators of adaptive potential: Clonal richness, inbreeding coefficient *F*_IS_, and LD. Temporal changes in these indicators, along with trends in overall genetic variation and connectivity can be assessed using informative and diagnostic SNPs for clone identification and differentiation of main genetic groups. These SNPs can be extracted from genome‐wide sequencing data and used as a SNP panel (or SNP chip). Such panels are already commonly used in research and can be successfully used in management (Andersson et al. 2024). A SNP panel is an affordable and simple tool for a managing authority, and all processing of tissue samples, DNA extractions, and genotyping may be outsourced. Furthermore, the generated data are less complex, requiring no further bioinformatic filtering or variant‐calling steps, and this approach can be used across geographic areas, enabling effective data combination or comparison (Andersson et al. 2024).

## 5 Conclusion

Using an example of a common species of macroalgae, *F. vesiculosus* , we have shown that in a coastal area with steep environmental gradients and species distributions influenced by postglacial colonisation processes, population genetic structures and phylogenetic relationships can be complex. Such complexity has both positive and negative implications for management and conservation. A positive implication of this is that high standing genetic variation enhances the adaptive potential during periods of rapid environmental changes. However, the negative side is that this complexity poses challenges in identifying and prioritising populations for conservation. High‐resolution genome‐wide genotyping offers the power to resolve these challenges and provide the analytical tools to develop a simple and relatively inexpensive genetic monitoring strategy. The first step involves mapping the genetic structure with high spatial coverage to identify pockets of unique genetic variation, “melting pots” and hybrid zones that can generate evolutionary novelties. Partial clonality adds complexity, particularly as sexually and asexually reproducing groups often form mosaic distributions. Therefore, integrating genetic data into monitoring programs for key species is critical to prevent the unnoticed loss of genetic variation essential for adaptation.

## Author Contributions

R.T.P. and K.J. conceptualised and conceived the study. R.T.P., A.K., A.L.M., O.O.‐M., P.R.J., S.P., M.I.M.P., M.T., P.D.W., C.A., H.K., and K.J. contributed the material and performed research. R.T.P., A.K., A.L.M., P.R.J., S.P., and M.I.M.P. analysed data. K.J. wrote the paper with input from R.T.P., A.K., and P.R.J. All co‐authors commented and approved the manuscript.

## Conflicts of Interest

The authors declare no conflicts of interest.

## Benefit Sharing Statement

A research collaboration was developed with scientists from the countries providing genetic samples. All collaborators are included as co‐authors or named in the acknowledgement. The results of research are shared with the provider communities and the broader scientific community through open‐access publication and data deposited in public databases (see above), and the research addresses a priority concern, in this case, the conservation of organisms being studied. More broadly, our group is committed to international scientific partnerships, as well as institutional capacity building, and joint ownership of relevant intellectual property rights.

## Supporting information


Data S1.

## Data Availability

Morphological and population genomic data are deposited in Dryad repository (DOI: 10.5061/dryad.q83bk3jsh). Scripts from the bioinformatic analysis are available at GitHub (https://github.com/crustaceana/Population‐genomics‐for‐Clonal‐organisms). Sequence data are available from NCBI SRA under BioProject accession PRJNA629489.
