# Supplementary material for: An Evolutionary Mosaic Challenges Traditional Monitoring of a Foundation Species in a Coastal Environment—The Baltic Fucus vesiculosus
Source: Mol Ecol. 2025 Feb 17;34(23):e17699. doi: 10.1111/mec.17699 (PMC12684334; doi:10.1111/mec.17699)
Supplement: Supplementary file 1 — Data S1. [file MEC-34-e17699-s001.pdf]

MOLECULAR ECOLOGY

Supplemental Information for:

An evolutionary mosaic challenges traditional monitoring of a foundation species in a coastal environment - the Baltic *Fucus vesiculosus*

Ricardo T. Pereyra, Alexandra Kinnby, Alan Le Moan, Olga Ortega-Martinez, Per Jonsson, Stefania Piarulli, Matthew I. M. Pinder, Mats Töpel, Pierre De Wit, Carl André, Halvor Knutsen, Kerstin Johannesson

Table of Contents:

**List of sampling sites (Table S1)**	Page 2
**Biophysical model of seascape connectivity**	Page 4
**Results of the demographic modelling (Tables S2 & S3)**	Page 6

MOLECULAR ECOLOGY

Table S1. List of sampling sites. Site no. 36/37 is a sympatric site in which both *Fucus vesiculosus* (sample 36) and *Fucus* sp. (sample 37) were sampled. In site no. 40/41 two samples were from very nearby sites (same long and lat). In sites no. 45, 46, 47 and 49, both sexually and clonally recruited individuals were sampled, as indicated by two different sample size numbers (N).

Site no	Country	Area	Locality	Long	Lat	N
1	France	Atlantic Ocean	Roscoff	3.990306 W	48.72569	14
2	UK	Atlantic Ocean	Bangor	4.130942 W	53.23381	11
3	Norway	Atlantic Ocean	Ålesund	6.5936	62.3352	6
4	Norway	Atlantic Ocean	Sandestrand	5.5830	59.0157	6
5	Norway	Atlantic Ocean	Skadbergsanden	5.9113	58.4566	6
6	Norway	Atlantic Ocean	Østhossel	6.6684	58.0749	4
7	Norway	Atlantic Ocean	Lillesand	8.3534	58.2242	13
8	Norway	Atlantic Ocean	Tvedestrand	8.9549	58.6104	18
9	Norway	Atlantic Ocean	Færder	10.4339	59.1394	18
10	Norway	Atlantic Ocean	Sandefjord	10.2247	59.0787	21
11	Germany	Atlantic Ocean	Sylt	8.43892	55.02092	17
12	Sweden	Atlantic Ocean	Tjärnö	11.13124	58.86678	15
13	Sweden	Atlantic Ocean	Kristineberg	11.44767	58.24773	12
14	Sweden	Atlantic Ocean	Espevik	12.18487	57.18986	18
15	Denmark	Transition zone	Bønnerup	10.71214	56.53311	15
16	Sweden	Transition zone	Vejbystrand	12.76233	56.31566	8
17	Denmark	Transition zone	Ebeltoft	10.66798	56.19467	17
18	Denmark	Transition zone	Juelsminde	10.00844	55.71683	10
19	Denmark	Transition zone	Kolding	9.62606	55.51372	20
20	Denmark	Transition zone	Helsingør	12.53644	55.954	18
21	Sweden	Transition zone	Landskrona	12.8099	55.8770	18
22	Sweden	Transition zone	Falsterbo	12.9341	55.4131	16
23	Denmark	Transition zone	Stege	12.17081	54.98386	17
24	Denmark	Transition zone	Høruphav	9.8855	54.90556	10
25	Germany	Transition zone	Kiel	10.18825	54.41169	15
26	Germany	Transition zone	Neustadt	10.80459	54.09126	16
27	Germany	Transition zone	Sassnitz	13.62706	54.39931	11
28	Sweden	Baltic Proper	Kivik	14.23059	55.68783	16
29	Sweden	Baltic Proper	Ottenby (Öland)	16.40004	56.19481	16
30	Sweden	Baltic Proper	Borgholm (Öland)	16.72077	56.88585	19
31	Sweden	Baltic Proper	Västervik	16.73196	57.6953	19
32	Latvia	Baltic Proper	Tuja	24.37959	57.48248	20

33	Estonia	Baltic Proper	Kõiguste (Saaremaa)	22.98222	58.37028	20
34	Estonia	Baltic Proper	Panga Pank (Saaremaa)	22.29861	58.56889	20
35	Estonia	Baltic Proper	Pulli Pank (Saaremaa)	22.95372	58.61473	20
36/37	Estonia	Baltic Proper	Sarve (Hiiumaa)	23.06278	58.84361	16+20
38	Sweden	Baltic Proper	Östernäs	18.99462	59.70227	13
39	Finland	Baltic Proper	Hankö	23.14083	59.83222	20
40/41	Finland	Gulf of Bothnia	Skärgårdshavet	21.83467	60.291	18+20
42	Sweden	Gulf of Bothnia	Djursten	18.40115	60.3687	20
43	Finland	Gulf of Bothnia	Rauma	21.3050	61.1435	20
44	Finland	Gulf of Bothnia	Björneborg	21.34905	61.48	29
45	Sweden	Gulf of Bothnia	Kuggören	17.51643	61.69878	20+19
46	Finland	Gulf of Bothnia	Sälskäret	21.2154	62.33361	20+19
47	Finland	Gulf of Bothnia	Storskäret	21.13582	62.47456	20+20
48	Sweden	Gulf of Bothnia	Barstahamn	18.40207	62.861	16
49	Finland	Gulf of Bothnia	South Vallgrund	21.38194	63.33694	11+19
50	Finland	Gulf of Bothnia	Hällkalla	21.08962	63.30737	12
51	Sweden	Gulf of Bothnia	Järnäs	19.66628	63.43556	8
52	Estonia	Gulf of Finland	Pakrineeme	24.13362	59.36889	20
53	Finland	Gulf of Finland	Helsinki	24.91765	60.14057	20
54	Estonia	Gulf of Finland	Letipea	26.61055	59.55205	18
55	Finland	Gulf of Finland	Kotka	26.950	60.470	8
56	Russia	Gulf of Finland	Primorsk	28.63007	60.36555	16

MOLECULAR ECOLOGY

Biophysical model of seascape connectivity

Dispersal and seascape connectivity was simulated with a biophysical model framework based on a Lagrangian particle-tracking model, TRACMASS (De Vries & Döös 2001), driven off-line with flow velocity fields from the ocean circulation model NEMO-Nordic (Hordoir et al., 2019). The ocean model grid has a horizontal spatial resolution of 3.7 km (2 NM) with 84 vertical levels, a free surface allowing the grid boxes to stretch and shrink vertically to allow for tides, and the atmospheric forcing is based on reanalysis of the ERA40 dataset (Uppala et al., 2005). At the model boundary, tidal harmonics define the sea surface height, and Levitus climatology defines temperature and salinity (Levitus & Boyer 1994). Climatological data for the Baltic Sea and the North Sea provided freshwater runoff. Validation of the NEMO-Nordic model has shown that the biophysical model is able to correctly represent the sea surface height, both tidally induced, and wind driven (Hordoir et al., 2019).

The biophysical model explored the multi-generational stepping-stone connectivity of *Fucus vesiculosus* to identify possible barriers to gene flow in the study area (Jahnke & Jonsson 2022). Specifically, the particle-tracking model was parameterised with drift depth at the surface (0-2 m), two drift durations (10 and 20 days with weights 90 and 10% respectively), and with particle release at mid-month according to the weights: May 30%, June 20%, July 5%, August 5%, September 30%, and October 10%. Model results are averages over 8 years (1995-2002) representing the range of the North Atlantic oscillation cycle (Hurrell & Deser 2009) that correlates well with large-scale circulation patterns in this region.

Particles were released from the 3,117 model grid cells in the Baltic that were considered as habitat for *Fucus vesiculosus* according to a species distribution model (Törnqvist et al., 2019). Briefly, the probability of presence of *Fucus vesiculosus* was based on occurrence data collected from 5 available databases and correlation to 12 environmental layers, including bathymetry, temperature, salinity, wave exposure, transparency and seabed substrate. The R-package BIOMOD2 (Guisan et al. 2017) was used to predict occurrence from a model ensemble. We used a threshold of 60% presence probability that best correlated to available field data on *Fucus vesiculosus* occurrence (AUC >0.8). The species distribution model has a resolution of 250 m and all ocean model grid cells (3.7 km) that contained any prediction of *Fucus vesiculosus* presence were considered as potential *Fucus* habitat, in total 3117 ocean grid cells.

Dispersal probabilities between all 3,117 grid cells were calculated and summarised in a connectivity matrix (Jonsson et al., 2020). From the connectivity matrix we calculated the multi-generation connectivity within the *Fucus* habitat assuming stepping-stone dispersal within the model domain. Multi-generation connectivity was calculated by multiplication of the connectivity matrix across 32 generations (Jahnke & Jonsson 2022). Finally, the multi-generation connectivity between the 50 sampling sites was presented as heatmaps. The estimates

of multi-generation connectivity should be interpreted as a relative measure since they are not scaled against site-specific reproductive output, which remains unknown.

References

De Vries, P., & Döös, K. (2001). Calculating Lagrangian trajectories using time-dependent velocity fields. *Journal of the Atmospheric Sciences*, *18*, 1092-1101.

Guisan, A., Thuiller, W., & Zimmermann, E. (2017). Habitat suitability and distribution models. Cambridge University Press.

Hordoir, R., Axell, L., Höglund, A., Dieterich, C., Fransner, F., Gröger, M., . . . Haapala., J. (2019). Nemo-Nordic 1.0: a NEMO-based ocean model for the Baltic and North seas – research and operational applications. *Geoscientific Model Development, 12*, 363-386.

Hurrell, J. W., & Deser, C. (2009). North Atlantic climate variability: The role of the North Atlantic Oscillation. *Journal of Marine Systems, 78*, 28-41.

Jahnke, M. & Jonsson, P. R. (2022). Biophysical models of dispersal contribute to seascape genetic analyses. *Philosophical transactions of the Royal Society of London. Series B, Biological sciences*, *377*, 20210024.

Jonsson, P. R., Moksnes, P. O., Corell, H., Bonsdorff, E., & Nilsson Jacobi, M. (2020). Ecological coherence of Marine Protected Areas: New tools applied to the Baltic Sea network. *Aquatic Conservation: Marine and Freshwater Ecosystems, 30*, 743-760.

Levitus, S., & Boyer, T. P. (1994). *World ocean atlas, vol 5, salinity*: NOAA atlas.

Törnqvist, O., Jonsson, P.R. & Hume, D. (2019). Climate refugia in the Baltic Sea: modelling future important habitats by using climate projections. Pan Baltic Scope Project. Available at: https://maritime-spatial-planning.ec.europa.eu/practices/climate-refugia-baltic-sea-modelling-future-important-habitats-using-climate.

Uppala, S. M., Källberg, P. W., Simmons, A. J., Andrae, U., Da Costa Bechtold, V., Fiorino, M., . . . Woollen, J. (2005). The ERA-40 re-analysis. *Quarterly Journal of the Royal Meteorological Society, 131*, 2961-3012.

MOLECULAR ECOLOGY

Results of the demographic modelling.

Table S2. Demographic modelling results for the best replicate (lower AIC value) of each of the 22 tested scenarios. The table shows, in order of appearance, the model tested, the AIC of the model, the differences in AIC with the overall best model (lowest AIC = SC2m model), the population mutation rate of the ancestral population (Θ), and the following parameter estimated by the model that are scaled to Θ: the change in ancestral population size, the population size of the first population (Ne_1 = Site 36), and second population (Ne_2= Site 37) after the split, the exponential population growth parameter in Site 36 (b_1) and Site 37 (b_2), the Hill-Robertson effect (hrf) corresponding to the fraction by which Ne is reduced in regions strongly affected by selection at linked sites, the time at which ancestral variation in effective size occurred (T_a), the time at which derived populations have split (T_s), the time of secondary contact or arrest of migration (T_{sc}/T_{am}), the effective migration rate ($M_{1>2}$ and $M_{2>1}$), the reduced effective migration rate ($Mr_{1>2}$ and $Mr_{2>1}$), and the fraction of the genome affected by reduced Ne or reduced migration rate (Q/P).

model	AIC	dAIC	Θ	Ne_a	Ne_1	Ne_2	b_1	b_2	hrf	T_a	T_s	T_{sc}/T_{am}	$M_{1>2}$	$M_{2>1}$	$Mr_{1>2}$	$Mr_{2>1}$	Q/P
SI	3251	1786	4099	2,807	0,089	0,142				1,965	0,016						
SI2N	2193	728	4859	2,623	0,153	0,240			0,192	1,251	0,009						0,560
SI2NG	2188	724	3027	4,043	0,170	0,404	0,344	0,170	0,222	3,049	0,007						0,499
SIG	3260	1795	1387	8,206	0,433	0,933	0,514	0,285		8,474	0,054						
IM	2005	540	1914	27,715	0,901	1,261				3,618	0,667		1,432	1,164			
IM2N	1488	23	2164	8,918	2,094	2,632			0,219	3,796	0,295		1,620	1,549			0,669
IM2NG	1531	66	3697	9,234	1,252	3,408	0,516	0,221	0,290	1,463	0,326		3,563	2,886			0,527
IM2m	1579	115	3176	30,573	0,623	0,793				1,594	0,437		0,740	0,841	3,953	3,784	0,354
**IM2mG**	**1471**	**6**	**302**	**38,494**	**3,514**	**0,807**	**0,331**	**2,023**		**18,638**	**2,015**		**12,819**	**5,142**	**0,366**	**1,025**	**0,934**
IMG	1973	508	2203	14,372	0,737	0,543	1,163	3,438		3,263	0,461		1,449	1,209			
AM	2029	565	432	84,845	4,286	5,944				20,000	0,042	3,040	0,328	0,255			
AM2N	1492	27	4039	5,753	1,337	1,909			0,127	1,442	0,096	0,003	5,669	4,490			0,792
AM2NG	1491	27	2317	7,753	1,599	1,649	1,937	4,236	0,182	3,590	0,236	0,007	1,734	1,300			0,712
AM2m	1596	131	2117	20,867	0,992	1,388				3,014	0,735	0,002	0,441	0,487	2,779	1,938	0,345
AM2mG	1619	154	2435	11,858	0,303	0,431	4,682	4,982		2,911	0,354	0,008	0,178	0,150	2,280	1,535	0,170
AMG	1955	490	1900	16,673	9,291	0,004	8,991	23,243		7,712	2,455	0,001	6,224	1,693			
SC	1989	524	2063	56,360	0,935	1,423				3,065	0,416	0,453	1,545	1,081			
SC2N	1526	62	1383	16,153	1,661	2,280			0,294	6,532	0,073	0,460	1,250	1,045			0,401
SC2NG	1485	21	1839	10,943	2,992	2,447	0,252	1,283	0,313	4,611	0,303	0,231	2,256	1,259			0,538
**SC2m**	**1465**	**0**	**1791**	**11,621**	**1,251**	**1,655**				**4,336**	**0,526**	**0,183**	**0,449**	**0,580**	**3,225**	**2,527**	**0,361**
SC2mG	1565	101	2408	11,833	1,907	0,125	0,141	28,412		3,074	0,012	0,234	0,889	0,790	3,457	5,256	0,367
SCG	1958	493	3132	12,762	1,816	1,263	0,178	0,560		1,286	0,806	0,202	2,553	2,821			

MOLECULAR ECOLOGY

Table S3. Transformation of the relevant parameters estimated from the two best-supported models (SC2m and IM2mG) and the next two best models (SC2NG and IM2N). Transformation was based on the method described in Rougeux et al. (2018), using a subset of 10,985 unlinked SNPs selected from 40,894 linked-SNPs detected from 17,043 RADtags of 36 base-pairs. We used a mutation rate of 1 x 10^{-8} and a generation time of 4 years to transform the parameters. In order of appearance, the table show the ancestral population size before (Nref) and after (Na) the ancestral change in effective size, the population size of population 1 (Ne_1; Site 36) and 2 (Ne_2; Site 37) after the split, the population size of population 1 (Ne_1 after exp.) and 2 (Ne_2 after exp.) after the exponential growth, the time of ancestral population growth (Ta), the time of split (Ts), the time of secondary contact (Tsc). Ne is estimated in number of individuals, and times are estimated in years.

Model	Nref	Na	Ne_1	Ne_2	Ne_1 after exp.	Ne_2 after exp.	Ta	Ts	Tsc
SC2m	271574	3155971	339642	449517	--	--	9420755	1142784	395412
SC2NG	278852	3051483	834326	682352	1073441	3009788	10286312	675936	515316
IM2mG	45752	160781	36902	1761187	51360	13314094	6821801	737561	--
IM2N	328133	2926291	687110	863646	--	--	9964748	774392	--
